# Alcohol expenditure in grocery stores and their associations with tobacco and food expenditures

**DOI:** 10.1186/s12889-019-7096-3

**Published:** 2019-06-20

**Authors:** Liisa Uusitalo, Maijaliisa Erkkola, Tomi Lintonen, Ossi Rahkonen, Jaakko Nevalainen

**Affiliations:** 1 Department of Food and Nutrition, FIN-00014 University of Helsinki, P.O. Box 66, Agnes Sjöbergin katu 2, Helsinki, Finland; 2 The Finnish Foundation for Alcohol Studies, c/o THL, P.O. Box 30, FIN-00271 Helsinki, Finland; 3 Department of Public Health, FIN-00014 University of Helsinki, P.O. Box 20, Tukholmankatu 8 B, Helsinki, Finland; 4 Health Sciences, Faculty of Social Sciences, FIN-33014 Tampere University, Tampere, Finland

**Keywords:** Alcoholic beverages, Purchase data, Health behaviour, Diet, Tobacco smoking

## Abstract

**Background:**

Alcohol consumption is a significant cause of disease, death and social harm, and it clusters with smoking tobacco and an unhealthy diet. Using automatically registered retail data for research purposes is a novel approach, which is not subject to underreporting bias. Based on loyalty card data (LoCard) obtained by a major Finnish retailer holding a market share of 47%, we examined alcohol expenditure and their associations with food and tobacco expenditures.

**Methods:**

The data consisted of 1,527,217 shopping events in 2016 among 13,274 loyalty card holders from southern Finland. A *K*-means cluster analysis was applied to group the shopping baskets according to their content of alcoholic beverages. The differences in the absolute and relative means of food and tobacco between the clusters were tested by linear mixed models with the loyalty card holder as the random factor.

**Results:**

By far, the most common basket type contained no alcoholic beverages, followed by baskets containing a small number of beers or ciders. The expenditure on food increased along with the expenditure on alcoholic beverages. The foods most consistently associated with alcohol purchases were sausages, soft drinks and snacks. The expenditure on cigarettes relative to total basket price peaked in the mid-price alcohol baskets.

**Conclusion:**

Clustering of unhealthy choices occurred on the level of individual shopping events. People who bought many alcoholic beverages did not trim their food budget. Automatically registered purchase data provide valuable insight into the health behaviours of individuals and the population.

**Electronic supplementary material:**

The online version of this article (10.1186/s12889-019-7096-3) contains supplementary material, which is available to authorized users.

## Background

Alcohol consumption is a significant cause of disease, death and social harm in most countries [1]. Alcohol use tends to cluster with tobacco smoking and unhealthy diets [2, 3], exacerbating inequalities in health. It is difficult to measure alcohol consumption reliably. Traditional survey methods are prone to memory errors and underreporting bias [4]. Using automatically registered purchase data to track retail purchases provides a novel approach, which has already been proven feasible in dietary studies [5–9].

Studies using large retail purchase datasets to reflect alcohol consumption and its associations with other health behaviours related to the purchase of products for consumption, such as healthy and unhealthy foods, are scarce. Individual transactions in supermarkets have been analysed in Denmark [10], with the aim of comparing the diets of wine and beer purchasers. Tobacco purchases were not reported. Sales data from a French supermarket chain were used to analyse associations of alcohol and foods, aggregating the purchases over a period of 1 year [11]. In a sample of British households, alcohol purchases were studied using two-week diaries kept by the participants [12]. Two studies conducted in the United States used product codes from alcohol and food packages that were self-scanned by the participants [13, 14].

The aim of this study is to analyse alcohol purchase patterns from grocery stores of a major Finnish retail chain on the level of individual shopping occasions. In 2016 when the data were collected, alcoholic beverages (beer, cider and alcopops) containing ≤4.7% alcohol were sold in grocery stores in Finland. In this exceptionally large data set, we analysed which alcoholic beverages, and in what quantities, were included in typical shopping baskets and if the alcoholic content of the shopping baskets was associated with food and tobacco expenditures.

## Methods

### Study sample

This study uses loyalty card data provided by the S Group (S-ryhmä), a major Finnish retailer co-operative. A detailed description of the study design and data can be found in [15]. The owners of S Group loyalty cards from a defined region in southern Finland were contacted via e-mail and asked for electronic informed consent to obtain selected background characteristics (age, gender and residential postal code) and purchase data from 1 January to 31 December, 2016, for research purposes, without personal identifiers. The invitation to the study was emailed to 245,877 customer owners, of which 13,274 (5.4%) gave their informed consent. Of them, 8937 (67%) were female and 4336 (33%) were male. The mean age was 46.2 years with a standard deviation of 14.7 years and a range from 16 to 90 years. The data consisted of 1,527,217 individual shopping events.

### Alcohol, tobacco and food variables

The information on the expenditures during each shopping occasion was in euros. We did not have explicit information about the purchased amounts in volume, weight or number of packages. For the purpose of illustrating the alcohol content of the shopping baskets, the approximate volumes were estimated on the basis of typical prices of alcoholic beverages in S group supermarkets. The alcohol variables used in the analyses were beer; cider, wine and alcopops containing ≤4.7% alcohol (the legal limit for alcoholic beverages sold in grocery stores in Finland 2016); and non-alcoholic beer, wine and cider (hereafter referred to as beer, cider and non-alcoholic options, respectively). Tobacco products were grouped to cigarettes, cigars and other tobacco products. The original grouping of 132 foods, provided by the S group, was based on ingredients, nutritional content and also on the package form and placement in the shelf system of the store (e.g. frozen pastries vs pastries from the in-store bakery). The original variables were combined into 68 larger groups by a nutrition researcher (LU) to be used in data descriptions and statistical analyses. The regrouping was verified by the other authors (ME, TL, OR and JN). To assist in the regrouping, a list of product names (but not the sales figures) within each product group was available. Because the food variables are used in analysing both health behaviour and its socioeconomic aspects, we used several criteria in their grouping process: food price and status, its connections to lifestyle or life stage, and the occasion of use as well as its wholesomeness. For instance, canned fish was held separate from other fish products because of the presumed low status of typical canned fish products tuna and sardine. Tex-mex products were kept as a separate group because their consumption may be associated with special occasions like parties or social evenings. The food group variables were comprehensive and non-overlapping. The original and regrouped food variables are shown in Additional file 1.

### Statistical analyses

A *K*-means cluster analysis was conducted to organize the shopping baskets of individual purchase occasions into clusters according to their content of alcoholic beverages (including non-alcoholic options). Because of the large data size, the analysis was done in two phases. First, a *K*-means cluster analysis was run in a random subsample of the main data (*n =* 2040) and the cluster centres were saved. Second, the baskets in the main data were assigned to the cluster they were closest to. We ran the analysis with the number of clusters set at 2–10. After scrutiny of the results, we decided to use the 8-cluster result. Up to eight clusters, increasing the number of clusters produced a solution in which each cluster distinguished itself from the others and could be interpreted in a meaningful way.

When describing the age and gender distribution of the buyers, the baskets with no alcoholic beverages formed a reference group against which the baskets with alcoholic beverages were compared. The food and tobacco contents of the alcohol-based clusters were analysed both as absolute euro amounts and as percentages of total food and tobacco expenditures. The absolute amounts were standardized (by subtracting the mean and dividing by the standard deviation) to ensure comparability between foods bought in different euro amounts. The differences in the means of food and tobacco between the clusters were tested by linear mixed models with the alcohol cluster as the fixed factor and loyalty card holder as the random factor. Statistical analyses were performed using IBM SPSS Statistics for Windows, Version 22.0 (Armonk, NY: IBM Corp.)

## Results

Using the *K*-means cluster analysis, we identified eight clusters of shopping baskets based on the expenditure and type of alcoholic beverages (beer, cider and non-alcoholic options). The approximate amount of alcoholic beverages in each basket, the number of baskets in each cluster and the gender and age distribution of people who bought them are presented in Table 1.

**Table 1:** Characteristics of alcohol baskets and of the customers who bought them^a^ in the LoCard data

	Clusters of shopping baskets based on expenditure on alcohol								
	No alcoholic beverages	Two beers	Two ciders	Beer for 15€	Cider for 15€, some beer	Beer for 30€	Beer for 50€	Alcohol for almost 100€, half beer, half cider	Beer for almost 100€
Abbreviation	No-alco	Two-Beers	Two-Ciders	Beer-15	Cider-15	Beer-30	Beer-50	Alco-100	Beer-100
Approximate volume of alcoholic beverages^b^	–	2 * 500 ml	2 * 330 ml	12 * 330 ml	5 * 500 ml	15 * 500 ml	24 * 500 ml	40 * 500 ml	100 * 300 ml
Number of baskets (%)	1,315,219 (86.1)	120,578 (7.9)	38,687 (2.5)	35,859 (2.4)	11,863 (0.8)	4000 (0.3)	612 (0.04)	262 (0.02)	137 (0.01)
Gender, %									
-Men	32.2	52	28.6	56.8	29.5	57.6	58.2	50.0	73.0
-Women	67.8	48	71.4	43.2	70.5	42.4	41.8	50.0	27.0
Mean age, years (range)	45.9 (16–90)	48.7 (18–70)	44.7 (18–84)	47.8 (18–84)	44.1 (18–84)	49.0 (18–81)	49.6 (18–73)	44.9 (18–72)	49.3 (18–72)
Age category, years^c^									
≤ 34	27.3	18.4	27.7	17.8	26.2	17.8	16.2	24.8	19.7
35–46	24.3	23.6	26.4	26.5	28.7	23.4	22.4	23.7	19.0
47–58	24.5	31.1	27.2	33.7	29.5	31.1	34.6	36.6	32.8
≥ 59	23.9	26.8	18.7	22.0	15.6	27.8	26.8	14.9	28.5

^a^Each customer is counted as many times as she/he made purchases. n = 1,527,217 shopping occasions

^b^Estimates are based on typical prices of alcoholic beverages

^c^Categories are based on quartile distribution

By far, the most common basket type was that with no alcoholic beverages (No-alco), followed by baskets with two beers (Two-Beers) or ciders (Two-Ciders) and then with beer for about 15€ (Beer-15) (Table 1). The basket type with cider for about 15€ (Cider-15) was purchased less often than the Beer-15 basket. The proportion of baskets with the largest expenditure on alcoholic beverages [beer for about 30€ (Beer-30) and upwards] was small. The results indicated that larger purchases of cider were typically accompanied by beer purchases as well.

Men were overrepresented among those who bought baskets dominated by beer, compared with those who did not buy alcoholic beverages. The difference was most pronounced in the largest basket, beer for almost 100€ (Beer-100). Women were slightly overrepresented among buyers of the smaller cider baskets, Two-Ciders and Cider-15, while male preponderance was seen in the basket of alcohol for almost 100€, half beer, half cider (Alco-100), when compared with customers who did not buy alcohol.

The mean age of customers who bought beer-dominated baskets was higher than the mean age of those who did not buy alcohol. The age group of 47–58 years was overrepresented in buying the baskets with alcohol. The youngest age group, younger than 35 years, was underrepresented in beer-dominated clusters, while customers 59 years or older were underrepresented in the cider-based clusters.

**Fig. 1:**
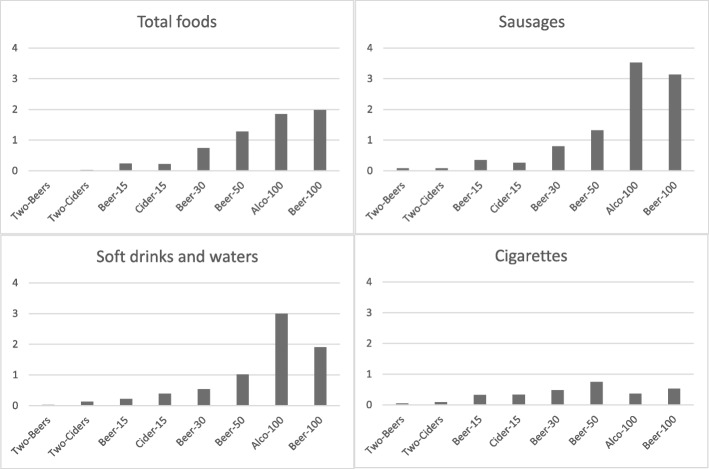
Bar charts showing the Z-scores of absolute euro amounts spent on total food, sausages, soft drinks and waters, and cigarettes from the LoCard purchase data (*n* = 1,527,217 shopping occasions)

**Fig. 2:**
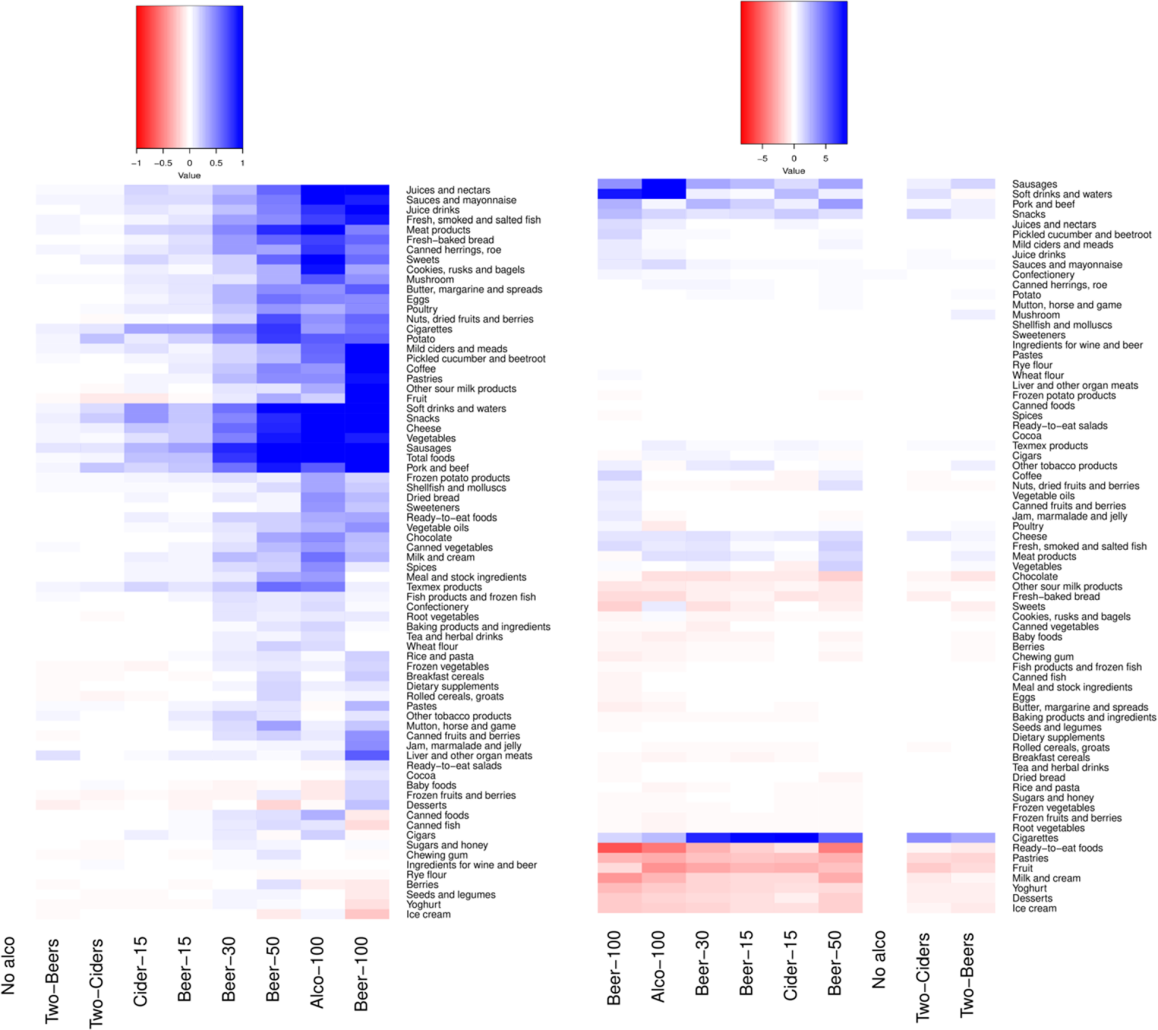
Heat map indicating the associations between alcohol and food purchases in the LoCard data (*n* = 1,527,217 shopping occasions). The differences in Z-scores of absolute euro amounts spent on each product group are given on the left panel, and the differences in the percentages of each product group of total purchases on the right panel, compared with the shopping baskets with no alcohol

The amount of money spent on food increased along with the total price of alcohol in the basket (Fig. 1). The smallest alcohol baskets, Two-Beers and Two-Ciders, were similar to No-alco baskets, both in the total amount spent on food and in the food groups chosen. In contrast, the food content of shopping baskets with larger expenditure on alcoholic beverages differed from No-alco baskets. Several foods were systematically associated with alcohol expenditure. The higher the total price of alcohol in the basket, the more money was spent on sausages, soft drinks and waters, snacks, cheese, juices and nectars, juice drinks, sauces and mayonnaise, and cookies, rusks and bagels (examples given in Fig. 1). Baskets with alcohol also contained more foods such as meats, eggs, fish, fats, milks, bread, vegetables, ready-to-eat foods and sweet foods than No-alco baskets (Fig. 2). However, a clear linear relationship between the amount of money spent on alcohol and on food was not observed. Likewise, cigarettes were bought more with alcohol, but the money spent on cigarettes was at its highest in the Beer-50 basket, followed by the Beer-100 and Beer-30 baskets.

There were some differences in food choices according to the alcoholic beverage bought (Fig. 2). Soft drinks and waters, cheese, sweets, cookies, rusks and bagels, and milk and cream were included in larger quantities in the baskets with cider as the dominating alcoholic beverage. On the contrary, fresh pork and beef, pastries, and pickled cucumber and beetroot were bought in larger quantities in baskets with beer as the dominating alcoholic beverage.

In addition to absolute amounts of money, we compared the proportion of each food from the total food and tobacco expenditures between the alcohol-based clusters of shopping baskets (Fig. 2). The percentage of money spent on sausages and snacks increased systematically as the alcohol basket grew larger, and there were up to threefold differences in the proportion of these foods between the alcohol baskets. The proportion of soft drinks and waters peaked in the two largest alcohol baskets, Alco-100 and Beer-100.

The proportion of money spent on fruit and ready-to-eat foods tended to be smaller in the larger alcohol baskets, but the difference was not significant in all baskets. The proportion of canned herrings and roe, pig and bovine meat, and mild ciders and meads tended to increase with increasing alcohol expenditure, but again, the difference was not significant in all baskets. The proportions of ice cream, desserts and yoghurt tended to be smaller in the four largest alcohol baskets, although the differences were not significant in the Alco-100 and Beer-100 baskets or for yoghurt in the Beer-50 basket.

The proportion of cigarettes was at its highest in the mid-sized alcohol baskets, Beer-15, Cider-15 and Beer-30.

## Discussion

In this study, alcohol purchase patterns and the clustering of alcohol with unhealthy foods and tobacco were described in a large data set of Finnish loyalty card holders. The typical expenditure on alcoholic beverages bought from grocery stores at one time was small. In the large majority of cases, no alcoholic beverages were bought, and less than 1 of 20 customers bought more than two beers or ciders. Beer was the most purchased type of alcoholic beverage, and cider was not bought in larger numbers without buying beer as well. Expenditures on non-alcoholic options were negligible; they were not characteristic to any of the clusters. The expenditure on alcohol were associated with expenditures on tobacco and foods rich in saturated fat, salt and added sugar.

Alcohol consumption and smoking tobacco are known to cluster together [2, 16]. However, their co-occurrence on the level of individual shopping occasions has not been studied before. The shopping basket clusters with alcoholic beverages contained more cigarettes than baskets without alcohol. However, cigarette expenditures did not peak in the baskets with the largest expenditure on alcoholic beverages but were most pronounced in the mid-sized alcohol baskets. It may be that the largest alcohol baskets were bought to be shared with many people at a social event, and cigarettes are not shared alongside alcohol. In addition, it is possible that people in whom unhealthy behaviours may cluster buy alcohol in moderate numbers but at a higher frequency. Socially disadvantaged people may buy their alcohol in smaller quantities at a time. To identify groups with potential problems with alcohol consumption, we will analyse the frequency and temporal distribution of alcohol expenditures in our next study.

The finding that the expenditure on alcoholic beverages was positively associated with the expenditure on food does not suggest that those who buy large numbers of alcoholic beverages would have to trim their food budget to compensate for the alcohol expenditure. However, only mild alcoholic beverages are allowed for sale in supermarkets and grocery stores in Finland, so wines and spirits were not included in our data. Mild alcoholic beverages cover about half of the total alcohol consumption in Finland [17]. Correspondingly, a similar association of less alcohol and smaller food budget was observed in French supermarkets using purchase data over 1 year [11], and the authors speculated that this could be explained by a lower socio-economic status. Over a two-week period, the expenditures for alcohol and food purchases in British households were inversely associated [12]. Our findings could indicate that the larger alcohol baskets were bought for social occasions and were intended to be shared with others. In fact, many of the food groups bought alongside the larger alcohol baskets – for instance, sausages, meats, soft drinks, snacks, cookies, sweets and pastries – are typical party or barbecue foods. The Danish study found that those who bought wine and beer also bought more food items [10]. Purchasing two different types of alcohol could indicate a social drinking occasion with the need for more food.

The food most consistently associated with alcohol expenditure in this study were sausages, soft drinks and snacks, as both the absolute and proportional expenditures on them grew consistently with the expenditure on alcoholic beverages. All three food categories may be seen as unhealthy options. Sausages and snacks tend to contain plenty of energy and fat, whereas soft drinks are major sources of refined sugar [18]. The aim of the Danish supermarket study was to compare food purchases of wine versus beer buyers [10]. The results showed that wine buyers favoured healthier foods, such as fruit and vegetables, poultry, oils and low-fat cheese, than beer buyers, who bought more sugar, chips, pork, lamb, sausages and soft drinks. In the French study in which purchases were aggregated over 1 year, beer buyers made unhealthier food choices than those who bought wine or did not buy alcoholic beverages [11]. Our results add to the earlier research that showed that alcohol consumption tends to cluster with unhealthy dietary choices. In many of the previous studies, a simpler indicator variable, such as fruit and vegetable consumption, or a pre-defined index of healthiness has often been used [2, 13, 19].

The same foods were associated with both beer- and cider-dominated shopping baskets, but expenditure on cider tended to correlate with sweet foods more strongly than expenditure on beer did. Perhaps people who choose cider prefer sweet tastes, or possibly this has something to do with the unequal gender or age distribution of cider versus beer buyers. Diets of people with different alcohol preferences have been compared before, mainly to account for the observed health benefits associated with wine [19, 20], but to our knowledge, comparisons between food choices of beer and cider drinkers have not been published before. In a French study, cider was aggregated with beer [21]. In light of these novel results, cider does not seem to associate with healthier food choices than beer like wine does.

This study, using automatically registered purchase data, has its unique strengths and weaknesses compared with traditional survey methods in alcohol and food research. A major weakness is that we did not know whether the foods and beverages were consumed by the same individual who bought them or if they were for family members or friends. Correspondingly, we did not know what was purchased elsewhere or eaten outside the home [10, 11, 13]. Wine and spirits are available only in specialist shops in Finland; therefore, a significant share of alcoholic beverages is outside the scope of our analyses. In general, however, food purchase data were seen to reflect individual diets reasonably well. The first analyses from this study indicate a very high proportion of expenditure among loyalty card owners in S Group stores [15]. The estimated mean of total annual expenditure in our data was 2322€ per customer, while the average consumption of groceries and non-alcoholic drinks in Finnish households was 2916€ per consumption unit during the same time period (22). Another limitation was that purchase data were on product group level and were imprecise for certain purposes. For example, we could not differentiate between common bulk beer and more exclusive craft beer varieties. The unit of measurement of expenditure was euros, and the actual weight or volume of the purchases was unknown.

A major limitation in this study, shared with survey samples, is the potential selection bias according to sociodemographic factors and alcohol consumption patterns. We know that women were over-represented in the present study population, compared with the gender distribution among residents of the study area, whereas both young and old people were under-represented [15]. The present study design offers no means to assess whether individuals who consume more alcohol were less likely to participate. However, it should be noted that the study was not presented to the loyalty card holders specifically as an alcohol consumption study, but as a study on purchase patterns at large. Therefore, it may be unlikely that alcohol consumption patterns as such would be the main driver of the decision of not to participate. Also, the consent for data use does not require personal contact with e.g. an interviewed, which may lower the “shame bound” for some individuals to release the details.

The major strengths of this study were large sample size, objective measurement of consumption without over- or underreporting [5, 10] and unbiased by recall or reporting error, and a potentially more heterogeneous study population than in traditional surveys [15]. The assessment of the association of food and alcohol on single shopping occasions would not be possible in survey data either. Due to social undesirability, self-reported alcohol consumption is typically underestimated. In this study, the permission for data use was requested from the participants in retrospect; thus, they were unaware of their status as study subjects at the time of data collection. As the study period covered a whole calendar year, the results were not affected by potential seasonal variation [10].

## Conclusions

In conclusion, clustering of unhealthy choices was seen on the level of individual shopping events, as alcohol expenditure coincided with expenditures on tobacco and foods with saturated fat, salt and added sugar. Numbers of alcoholic beverages bought from grocery stores at one time were small. When large amounts of alcoholic beverages were bought, the expenditure was not compensated by trimming the food budgetThe present study demonstrates the feasibility of analysing individual shopping occasions in large sales data to shed light on consumer choices connected with health behaviour. Automatically registered retail purchase data has its unique strengths and weaknesses compared with more traditional survey data, and as such it provides new points of view into population health behaviour, adding to knowledge based on other methods. The results can be used as an evidence base for targeting interventions and policies. For example, an electronic app providing automatic feedback to the customer based on the contents of individual shopping baskets seems feasible on the basis of the current results. Furthermore, large-scale, country-wide and timely expenditure data could serve as an alert system, should consumption patterns indicative of health risks emerge in certain premises, areas or subgroups.

## Additional file

Additional file 1: Regrouping of the original food variables in the purchase data of the LoCard project, 2016. (PDF 216 kb)

## Data Availability

Data are owned by a third party (S Group) and were used under a research agreement for the current study, and are not publicly available. According to the research agreement, the authors are not allowed to share the data.

## Abbreviations

Alco-100: Alcohol for almost 100€, half beer, half cider
Beer-100: Beer for almost 100€
Beer-15: Beer for 15€
Beer-30: Beer for 30€
Beer-50: Beer for 50€
Cider-15: Cider for 15€, some beer
No-alco: No alcoholic beverages
Two-Beers: Two beers
Two-Ciders: Two ciders

## References

[1] World Health Organization (2014). Global status report on alcohol and health 2014.

[2] Noble N, Paul C, Turon H, Oldmeadow C (2015). Which modifiable health risk behaviours are related? A systematic review of the clustering of smoking, nutrition, alcohol and physical activity ('SNAP') health risk factors. Prev Med.

[3] Falkstedt D, Moller J, Zeebari Z, Engstrom K (2016). Prevalence, co-occurrence, and clustering of health-risk behaviors among people with different socio-economic trajectories: a population-based study. Prev Med.

[4] Livingston M, Callinan S (2015). Underreporting in alcohol surveys: whose drinking is underestimated?. J Stud Alcohol Drugs.

[5] Tin ST, Mhurchu CN, Bullen C (2007). Supermarket sales data: feasibility and applicability in population food and nutrition monitoring. Nutr Rev.

[6] Smed S, Tetens I, Boker Lund T, Holm L, Ljungdalh Nielsen A (2018). The consequences of unemployment on diet composition and purchase behaviour: a longitudinal study from Denmark. Public Health Nutr.

[7] Lund TB, Watson D, Smed S, Holm L, Eisler T, Nielsen A (2017). The diet-related GHG index: construction and validation of a brief questionnaire-based index. Clim Chang.

[8] Becker W (2001). Comparability of household and individual food consumption data--evidence from Sweden. Public Health Nutr.

[9] Nelson M, Dyson PA, Paul AA (1985). Family food purchases and home food consumption: comparison of nutrient contents. Br J Nutr.

[10] Johansen D, Friis K, Skovenborg E, Gronbaek M (2006). Food buying habits of people who buy wine or beer: cross sectional study. BMJ.

[11] Hansel B, Roussel R, Diguet V, Deplaude A, Chapman MJ, Bruckert E (2015). Relationships between consumption of alcoholic beverages and healthy foods: the French supermarket cohort of 196,000 subjects. Eur J Prev Cardiol.

[12] Gell L, Meier P (2012). The nature and strength of the relationship between expenditure on alcohol and food: an analysis of adult-only households in the UK. Drug Alcohol Rev.

[13] Adjemian MK, Volpe RJ, Adjemian J (2015). Relationships between diet, alcohol preference, and heart disease and type 2 diabetes among Americans. PLoS One.

[14] Volpe R, McCullough M, Adjemian MK, Park T (2016). Beer-purchasing behavior, dietary quality, and healthoutcomes among U.S. adults. J Wine Econ.

[15] Nevalainen J, Erkkola M, Saarijarvi H, Nappila T, Fogelholm M. Large-scale loyalty card data in health research. Digit Health. 2018 Nov 29;4 2055207618816898. 10.1177/2055207618816898 PMC6287323 30546912

[16] Meader N, King K, Moe-Byrne T, Wright K, Graham H, Petticrew M (2016). A systematic review on the clustering and co-occurrence of multiple risk behaviours. BMC Public Health.

[17] Yearbook of Alcohol and Drug Statistics 2017. Helsinki, Finland: National Institute for Health and Welfare; 2107.

[18] Nordic Nutrition Recommendations 2012, 5th edition. 2014.

[19] Barefoot JC, Gronbaek M, Feaganes JR, McPherson RS, Williams RB, Siegler IC (2002). Alcoholic beverage preference, diet, and health habits in the UNC alumni heart study. Am J Clin Nutr.

[20] Sluik D, Bezemer R, Sierksma A, Feskens E (2016). Alcoholic beverage preference and dietary habits: a systematic literature review. Crit Rev Food Sci Nutr.

[21] Rouillier P, Boutron-Ruault MC, Bertrais S, Arnault N, Daudin JJ, Bacro JN (2004). Drinking patterns in French adult men--a cluster analysis of alcoholic beverages and relationship with lifestyle. Eur J Nutr.

