# Supplementary material for: Alcohol expenditure in grocery stores and their associations with tobacco and food expenditures
Source: BMC Public Health. 2019 Jun 20;19:787. doi: 10.1186/s12889-019-7096-3 (PMC6587280; doi:10.1186/s12889-019-7096-3)
Supplement: Supplementary file 1 — Regrouping of the original food variables in the purchase data of the LoCard project, 2016. (PDF 216 kb) [file 12889_2019_7096_MOESM1_ESM.pdf]

Additional file 1: Regrouping of the original food variables in the purchase data of the LoCard project, 2016

**Food groups used in analyses**	**Original food variables**
Baby foods	Baby foods
Baking products and ingredients	Ingredients for baking and gelatinizing, breadcrumbs
	Other flours and ingredients
	Preservatives
	Starches
	Baker's yeast
Beef and pork	Beef
	Pork
Berries	Forest berries
	Garden berries and rhubarb
Breakfast cereals	Breakfast cereals
Butter, margarine and spreads	Butter, margarine and spreads
Canned fish	Canned fish
Canned foods	Canned foods
Canned fruits and berries	Canned fruits and berries
Canned herrings, roe	Roe
	Canned herrings
Canned vegetables	Canned vegetables
Cheese	Cheese
Chewing gum	Chewing gum
Chocolate	Chocolate
Cocoa	Cocoa
Coffee	Coffee
	Instant coffee
Confectionery	Confectionery
Cookies, rusks and bagels	Cookies
	Rusks and bagels
Desserts	Desserts
	Milk-based desserts
Dietary supplements	Natural products VAT17 %
	Natural products VAT22 %
	Health salts
Dried bread	Dried bread
Eggs	Eggs
Fish products and frozen fish	Frozen fish and shellfish
	Other fish products
	Smoked fish
	Salted fish
	Fresh fish
Fresh-baked bread	Fresh-baked bread

Frozen fruits and berries	Frozen fruits, berries and juices
Frozen potato products	Frozen potato products
Frozen vegetables	Frozen vegetables
Fruit	Bananas
	Stone fruits
	Other fruits
	Apples
	Pears
	Grapes
	Citrus fruits
Ice cream	Ice cream
Jam, marmalade and jelly	Jam
	Marmalade and jelly
Juice drinks	Juice concentrates and powders, sports drinks
	Sweetened juice drinks
	Juice drinks
Juices and nectars	Juices and nectars
Liver and other organ meats	Organ meats
	Liver products
Meal and stock ingredients	Meal and stock ingredients
	Ingredients for mashed potato
Meat products	Meat products
	Frozen meat products
	Semi-finished meat products
	Meat preservatives
	Smoked meat products
Milk and cream	Milk and cream
	Milk and cream powder
Mushroom	Cultivated mushroom
	Forest mushroom
Mutton, horse and game	Mutton
	Other meat
	Game
Nuts, dried fruits and berries	Dried fruits and berries
	Nuts and almonds
Other sour milk products	Other sour milk products (than yoghurt)
Pastes	Pastes
Pastries	Pastries
	In-store bakery
	Frozen pastries
	Other bakery products
Pickled cucumber and beetroot	Pickled cucumber and beetroot
Potato	Potato
Poultry	Frozen poultry products

	Poultry
Ready-to-eat foods	Convenience foods
	Ready-to-eat bakery products
	Other ready foods
	Packed fast food
	Packed ready-to-eat-foods
	Ready-to-eat foods from service counter
	Frozen meals
Ready-to-eat salads	Packed ready-to-eat salads
	Ready-to-eat salads from service counter
Rice and pasta	Pasta
	Rice
Rolled cereals, groats	Rolled cereals
	Groats
Root vegetables	Root vegetables
Rye flour	Rye flour
Sauces and mayonnaise	Sauces and mayonnaise
	Mustard
Sausages	Cured sausages
	Cold-cut sausage bars
	Cold-cut sausage slices
	Sausages
Seeds and legumes	Other grain products
Shellfish and molluscs	Preserved shellfish and molluscs
	Shellfish and molluscs
Snacks	Snacks
Soft drinks and waters	Soft drinks and waters
Spices	Vinegars
	Spices
	Salts
Sugars and honey	Honey
	Treacle
	Sugars
Sweeteners	Sweeteners
Sweets	Sweets, special VAT 22%
	Liquorice
	Bags of candy
	Other sweets
	Candy boxes
Tea and herbal drinks	Tea and herbal drinks
Texmex products	Texmex products
Vegetable oils	Vegetable oils
Vegetables	Herbs and pulses
	Salads
	Salad mixes

	Vegetables
Wheat flour	Wheat flour
Yoghurt	Yoghurt
